# How to reconstruct the lordosis of cervical spine in patients with Hirayama disease? A finite element analysis of biomechanical changes focusing on adjacent segments after anterior cervical discectomy and fusion

**DOI:** 10.1186/s13018-022-02984-y

**Published:** 2022-02-16

**Authors:** Xiao Lu, Fei Zou, Feizhou Lu, Xiaosheng Ma, Xinlei Xia, Jianyuan Jiang

**Affiliations:** Department of Orthopedics, Huashan Hospital, Fudan University, No. 12, Middle Wulumuqi Road, Jing’an District, Shanghai, 200040 China

**Keywords:** Hirayama disease, Finite element analysis, ACDF, Biomechanics, Degeneration

## Abstract

**Purpose:**

To compare the biomechanical changes of adjacent segments between patients with Hirayama disease and non-pathological people after anterior cervical discectomy and fusion (ACDF) operation, and to explore the optimal degree of local lordosis reconstruction during surgery.

**Methods:**

A young male volunteer was recruited to establish a three-dimensional finite element model of the lower cervical spine based on the CT data. By adjusting the bony structures and simulating the operation process, the models of non-pathological individuals before and after ACDF, patients with Hirayama disease before and after ACDF, and different local lordosis angles were established. Then, the postoperative range of motion (RoM) and stress of the adjacent segments under flexion, extension, left bending, right bending, left rotation and right rotation were recorded and compared.

**Results:**

The RoM and stress of all segments of lower cervical spine in patients with Hirayama disease are higher than those in non-pathological individual, and this trend still exists after ACDF surgery. When the local lordosis angle is under physiological conditions, the RoM and stress of the adjacent segments are minimum.

**Conclusion:**

Compared with non-pathological people, Hirayama disease patients have differences in cervical biomechanics, which may lead to cervical hypermobility and overload. After ACDF, the possibility of adjacent segments degeneration is greater than that of non-pathological people. When the operation maintains the physiological local lordosis angle, it can slow down the degeneration.

## Introduction

Hirayama disease, also known as juvenile muscular atrophy of distal upper extremity, is a disease characterized by asymmetrical atrophy of the intrinsic muscles of the hand and forearm muscles. It occurs frequently in adolescents, with an average age of 15–20 years old [1, 2]. Its clinical manifestations are asymmetrical muscle atrophy and weakness in the distal part of the unilateral upper limb, with tremor and cold paralysis, no sensory disturbance and pyramidal tract damage [3, 4]. Hirayama disease mainly affects the hand function of teenagers, resulting in the decline or even loss of patients’ ability to work, which brings a heavy burden to individuals, families and society.

In the treatment of Hirayama disease, a neck brace can be worn at an early stage, but for those who cannot adhere to wearing it or whose disease course is rapidly progressing, anterior cervical discectomy and fusion (ACDF) surgery is considered one of the effective treatments. The purpose of the operation is to reconstruct the physiological curvature of the cervical spine, reduce the range of motion (RoM) of the cervical spine, and prevent the forward compression of the spinal cord in the flexion position. Both imaging and clinical scores have proved its effectiveness [5–7].

The bony structure of the cervical spine in Hirayama disease is different from that in non-pathological people, and the curvature of the cervical spine becomes straight or kyphosis [8, 9]. We found that when the cervical curvature was reconstructed to normal physiological curvature during ACDF, the upper adjacent segment would have compensatory kyphosis, so how to reconstruct the cervical curvature of Hirayama disease patients remains unclear. In this study, eight three-dimensional (3D) finite element models of non-pathological lower cervical spine (NLCS), NLCS + C4–6 ACDF, NLCS + C5–7 ACDF, lower cervical spine of Hirayama disease (LCSHD), LCSHD + C4–6 ACDF, LCSHD + C5–7 ACDF, LCSHD + C4–6 ACDF + C4–6 posterior wall angle (PWA) 0°, and LCSHD + C4–6 ACDF + C4–6 PWA 5° were established by 3D finite element analysis, and the biomechanical differences of adjacent segments of intervertebral discs between non-pathological people and patients with Hirayama disease after ACDF were compared.

The main purpose of this study was to compare the biomechanical changes of adjacent segments in Hirayama disease patients and non-pathological controls by 3D finite element analysis. Then, we explored how to reconstruct the cervical curvature of Hirayama disease patients through ACDF to minimize the impact on adjacent segments.

## Materials and methods

### Establishment of 3D finite element model

A healthy male volunteer, aged 24 years, with a height of 170 cm and a weight of 60 kg, was recruited. There was no previous history of neck disease. 64 slice CT (Siemen Company; Germany) was performed in the CT room of medical imaging center, Huashan Hospital, Fudan University (120 kV, 125 mA, scanning thickness 0.625 mm, range C2–T2). The CT scan data were exported and saved in DICOM format, and a total of 260 images were obtained. And the project was approved by the ethics committee of Huashan Hospital (KY-2019-546).

**Fig. 1:**
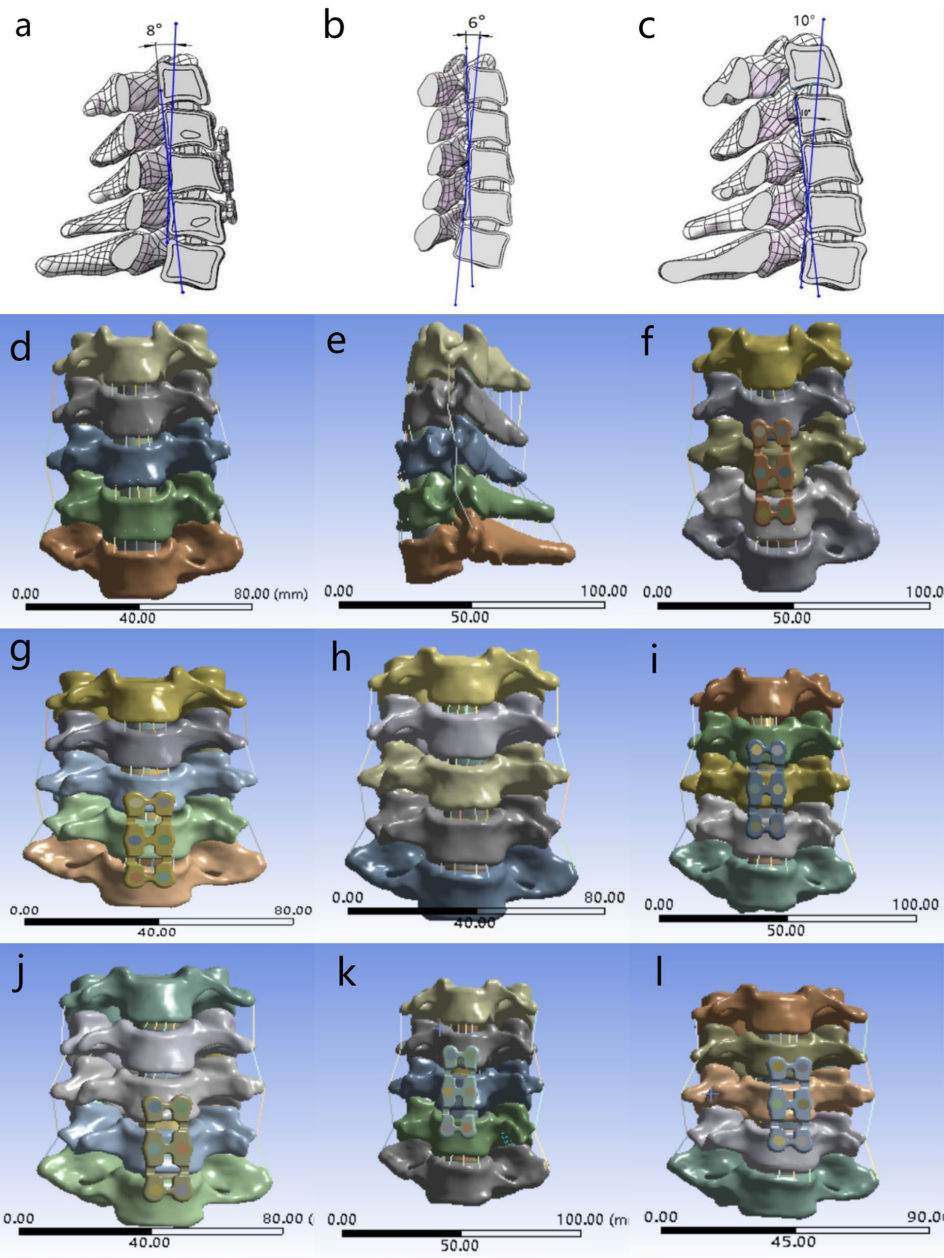
The three-dimensional finite element models established in this study. **a** The C4–6 PWA of healthy volunteer in this study was 8°. **b**, **c** Reduce or increase C4–6 PWA by 2°. **d**, **e** The front and side view of the model of NLCS. f-l.The front view of the model of NLCS + C4–6 ACDF (F), NLCS + C5–7 ACDF (**g**), LCSHD (**h**), LCSHD + C4–6 ACDF (**i**), LCSHD + C5–7 ACDF (**j**), LCSHD + C4–6 ACDF + C4–6 PWA 6°(**k**), and LCSHD + C4–6 ACDF + C4–6 PWA 10°(**l**). ACDF, anterior cervical discectomy and fusion; NLCS, non-pathological lower cervical spine; LCSHD, lower cervical spine of Hirayama disease; PWA, posterior wall angle

The CT data were imported into Mimics 21.0 (Materialise, Belgium) software to segment each vertebral body and establish the non-pathological lower cervical spine model of C3–C7. Then, the file was imported into Geomagic wrap 2017 (Geomagic company, USA) for polishing, smoothing and other processing to make its shape close to the bony structure of cervical spine. Next, it was imported into SolidWorks 2021 (Dassault Systems, USA) software to complete the solid model of each vertebral body. On this basis, two cartilage endplate models and a whole intervertebral disc model of adjacent vertebrae were reconstructed, in which the endplate thickness was set to 0.6 mm. According to the literature reports and anatomical data, the nucleus pulposus and annulus fibrosus were segmented at the proportion of 6:4 [10, 11]. Besides, the model of Hirayama disease was constructed by reducing the height of uncinate process and the inclination angle of inferior endplate [9] and increasing the angle of disc-facet [8] in the non-pathological model (Table 1). The posterior wall angle (PWA) of the vertebral bodies is defined as the angle between the posterior wall of C4 and the posterior wall of C6 in the sagittal position. The volunteers' PWA of C4–6 was 8°, and then, the local lordosis was reconstructed to 6 and 10 degrees to simulate the postoperative effect (Fig. 1). Finally, the whole model was imported into ANSYS 17.0 (ANSYS Company, USA) software, and the parameters provided by previous studies [11–13] were used to assign values to different material properties to establish a 3D finite element model of the lower cervical spine of C3–C7 (Table 2).

**Table 1:** The height of uncinate process, the inclination angle of inferior endplate, and the angle of disc-facet of non-pathological models and Hirayama disease models in this study and previous literature

Cervical vertebra	Non-pathological model of this study		Hirayama disease		
	Left	Right	Literature	Left of this study	Right of this study
*The height of uncinate process (mm)*					
C3	4.51	5.02	3.79 ± 1.16	3.79	3.79
C4	4.20	4.49	3.64 ± 1.04	3.64	3.64
C5	4.64	5.39	4.20 ± 0.77	4.20	4.20
C6	5.10	5.50	4.37 ± 1.01	4.37	4.37
C7	4.99	6.00	3.95 ± 1.13	3.95	3.95
*The inclination angle of inferior endplate (°)*					
C3	110.18	110.08	119.33 ± 11.93	119.33	119.33
C4	112.11	112.93	103.54 ± 4.10	103.54	103.54
C5	116.41	116.75	105.09 ± 3.90	105.09	105.09
C6	105.87	113.76	104.99 ± 3.33	104.99	104.99
C7	110.35	111.61	105.00 ± 4.81	104.99	104.99
*The angle of disc-facet (°)*					
C3	114.50	112.11	121.41 ± 7.31	121.41	121.41
C4	121.55	126.56	125.46 ± 5.59	125.46	125.46
C5	127.12	126.59	127.38 ± 6.47	127.38	127.38
C6	122.30	124.76	124.27 ± 6.76	124.27	124.27
C7	112.24	111.51	113.67 ± 5.94	113.67	113.67

**Table 2:** Material properties used in the finite element models

Materials	Elastic modulus/MPa	Poisson's ratio	Cross-sectional area (mm^2^)
Titanium plate, screw	105,000	0.342	–
Bone cortex	12,000	0.3	–
Cancellous bone	450	0.2	–
Articular cartilage	10.4	0.4	–
Endplate	500	0.4	–
Nucleus pulposus	1	0.49	–
Annular fibre	110	0.3	–
Annulus fibrosus matrix	3.4	0.4	6
Anterior longitudinal ligament	10	0.3	5
Posterior longitudinal ligament	10	0.3	46
Articular capsule ligament	10	0.3	5
Ligamentum flavum	1.5	0.3	10
Interspinous ligament	1.5	0.3	5
Supraspinous ligament	1.5	0.3	5
Intertransverse ligament	1.5	0.3	–

### Surgery simulation

In our clinical work, we selected single level or double levels ACDF for patients with Hirayama disease according to their condition. We found that the number of cases of double levels ACDF was the largest. Therefore, in order to make the model more representative, we simulated double levels surgery in this study. Taking the C4–6 segments as an example, the anterior longitudinal ligament was excised and the C4–6 intervertebral discs were scraped, then the C4–6 intervertebral discs and endplates were changed to cancellous bone based on the parameters in Table 2 to simulate the bone graft fusion state after ACDF. Finally, the operative segments were fixed with titanium plate. In the course of treatment, C4–6 and C5–7 are the most common surgical segments. According to this method, the finite element models of NLCS + C4–6 ACDF, NLCS + C5–7 ACDF, LCSHD + C4–6 ACDF, LCSHD + C5–7 ACDF, LCSHD + C4–6 ACDF + C4–6 PWA 6°, and LCSHD + C4–6 ACDF + C4–6 PWA 10° were established (Fig. 1).

### Boundary setting and loading conditions of 3D finite element model

In ANSYS16.0, the lower endplate of C7 vertebral body of the 3D finite element model was completely constrained and fixed. An axial load of 73.6 N was applied to the upper endplate of C3 vertebral body to simulate head weight. Then, the *X*, *Y*, *Z* global coordinate system was established, the *X*–*Z* plane was the coronal plane, the *X*–*Y* plane was the horizontal plane, and the *Y*–*Z* plane was the sagittal plane. The torque of 1 Nm was applied, and the torque direction was set according to the right hand rule. After loading, the model completed six movements: forward flexion, backward extension, left and right bending and left and right rotation.

### Observation index

The range of motion (RoM) and maximum stress of adjacent segments under flexion, extension, left bending, right bending, left rotation and right rotation were recorded. Then compared the changes of cervical motion and mechanical properties between Hirayama disease and non-pathological people after ACDF under different working conditions.

RoM refers to the range of motion of cervical spine in sagittal plane, coronal plane and cross section. It is one of the important indicators of cervical function, curative effect evaluation and prognosis analysis of cervical spondylosis. After applying the corresponding load and torque to the model by 3D finite element analysis software, the six working conditions of cervical motion were simulated to obtain the RoM of adjacent segments of the surgical site.

Disc stress alteration has a large effect on the biomechanical environment of the intervertebral space and the internal environment of the disc. The stress cloud diagram can directly display the stress and pressure in all areas of the whole model in different colours. The maximum von Mises stress of intervertebral disc is the most commonly used mechanical quantity in 3D finite element analysis of spine, which reflects the maximum stress area of intervertebral disc.

## Results

### Model validation

**Fig. 2:**
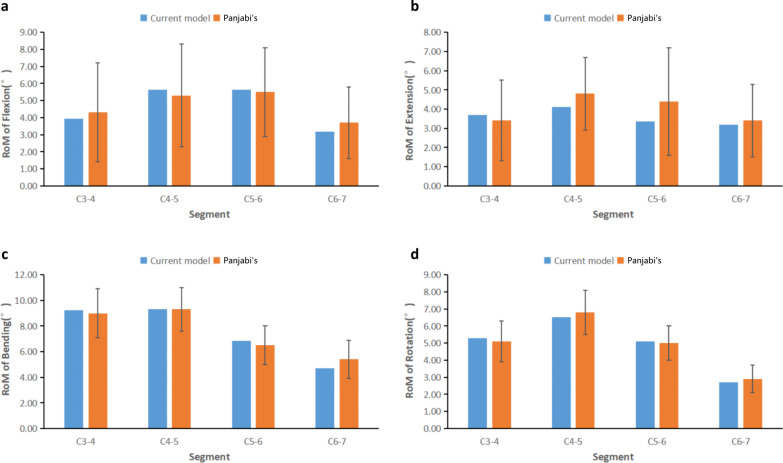
Validation of non-pathological lower cervical spine model. The range of motion of the model during flexion (**a**), extension (**b**), lateral bending (**c**) and rotation (**d**) is similar to that of previous studies, and the model is effective

The 3D finite element model of the non-pathological lower cervical spine built in this study involved 165,522 nodes and 87,716 cells. The model was realistic in appearance and contained important anatomical structures such as cervical vertebral bodies, transverse processes, articular processes, intervertebral discs and ligaments. The RoM of forward flexion, backward extension, lateral bending, and rotation was basically consistent with the data reported in the classic literature [14, 15] and could be used for biomechanical studies of the cervical spine. (Fig. 2).

### RoM and stress of non-pathological people and Hirayama disease patient

**Fig. 3:**
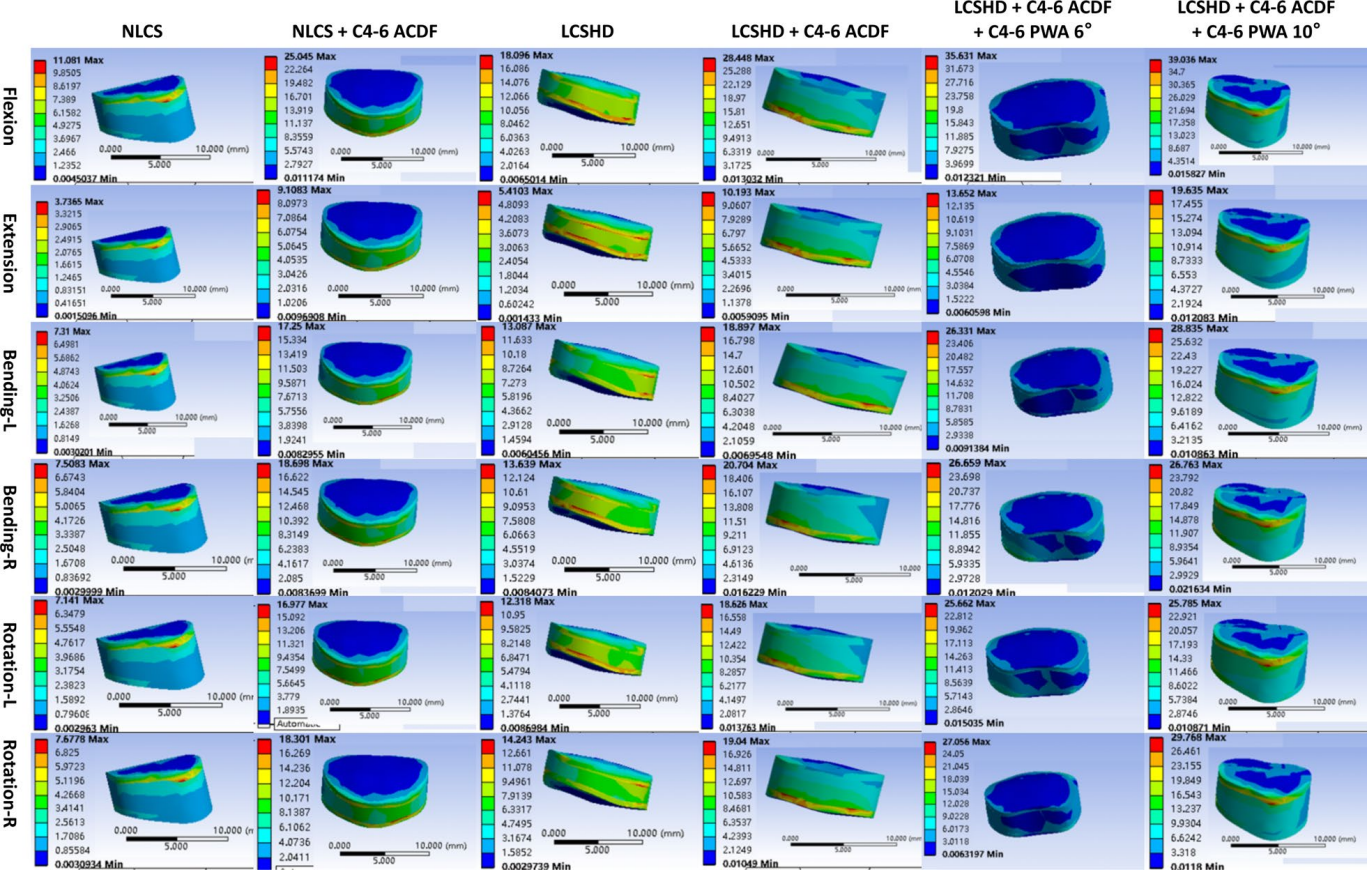
The maximum von Mises stress of C3–4 intervertebral disc. ACDF, anterior cervical discectomy and fusion; NLCS, non-pathological lower cervical spine; LCSHD, lower cervical spine of Hirayama disease; PWA, posterior wall angle

**Fig. 4:**
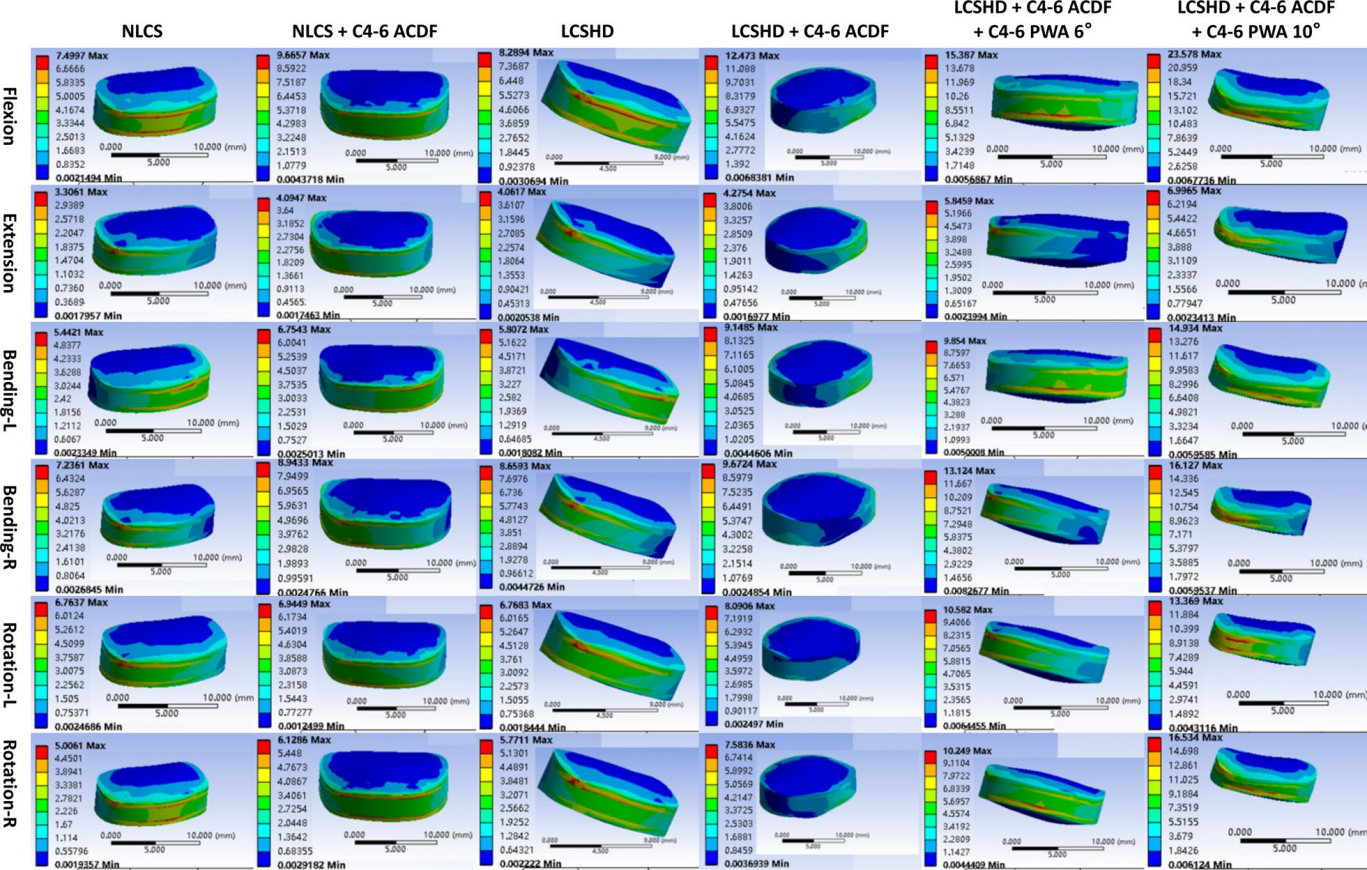
The maximum von Mises stress of C6–7 intervertebral disc. ACDF, anterior cervical discectomy and fusion; NLCS, non-pathological lower cervical spine; LCSHD, lower cervical spine of Hirayama disease; PWA, posterior wall angle

Compared with the results of non-pathological people and Hirayama disease, it could be easily found that the RoM and stress of all segments in patients with Hirayama disease were higher than that of non-pathological people (Table 3; Figs. 3, 4).

**Table 3:** RoM comparison of six models under six different working conditions

RoM of C3–4 (°)						
Working condition	NLCS	NLCS + C4–6 ACDF	LCSHD	LCSHD + C4–6 ACDF	LCSHD + C4–6 ACDF + C4–6 PWA 6°	LCSHD + C4–6 ACDF + C4–6 PWA 10°
Forward flexion	3.94	11.10	9.81	16.85	17.92	19.02
Backward extension	3.67	6.31	5.88	7.71	8.49	9.56
Left bending	9.22	14.55	15.22	17.77	19.48	20.32
Right bending	9.48	14.67	16.56	18.97	20.65	22.47
Left rotation	5.29	10.10	10.39	13.12	17.17	17.56
Right rotation	5.34	10.53	12.10	15.86	17.75	18.06

RoM of C4–5 (°)					RoM of C5–6 (°)	
Working condition	NLCS	NLCS + C5–7 ACDF	LCSHD	LCSHD + C5–7 ACDF	NLCS°	LCSHD
Forward flexion	5.64	7.04	6.16	8.38	5.63	5.83
Backward extension	4.12	4.53	4.30	5.35	3.36	3.41
Left bending	9.31	10.34	9.73	11.35	6.85	7.30
Right bending	9.54	10.44	9.82	11.48	7.26	7.82
Left rotation	6.51	7.46	6.76	8.47	5.10	5.59
Right rotation	6.43	7.41	6.77	8.33	5.00	5.31

RoM of C6–7 (°)						
Working condition	NLCS	NLCS + C4–6 ACDF	LCSHD	LCSHD + C4–6 ACDF	LCSHD + C4–6 ACDF + C4–6 PWA 6°	LCSHD + C4–6 ACDF + C4–6 PWA 10°
Forward flexion	3.17	5.41	3.43	5.70	6.52	7.02
Backward extension	3.18	3.72	3.66	3.97	4.56	4.63
Left bending	4.72	6.68	5.51	6.91	7.14	8.32
Right bending	4.74	6.66	5.31	6.84	7.23	8.47
Left rotation	2.70	3.96	3.06	4.50	5.07	6.56
Right rotation	2.70	4.23	2.87	4.75	4.80	6.06

### RoM and stress of adjacent segments after ACDF

After the operation of C4–6 segments, the RoM of non-pathological people in C3–4 segment was 11.10°, 6.31°, 14.55°, 14.67°, 10.10°and 10.53°, respectively, during flexion, extension, left bending, right bending, left rotation and right rotation. However, for Hirayama patient, the RoM under the six conditions was 16.85°, 7.71°, 17.77°, 18.97°, 13.12° and 15.86°. As for C6–7 segment, the RoM of non-pathological people under six conditions was 5.41°, 3.72°, 6.68°, 6.66°, 3.96° and 4.23°, while for Hirayama patient, those were 5.70°, 3.97°, 6.91°, 6.84°, 4.50° and 4.75° (Table 3).

The maximum von Mises stress of intervertebral disc in non-pathological model during six conditions was 25.05 MPa, 9.11 MPa, 17.25 MPa, 18.70 MPa, 16.98 MPa, and 18.30 MPa, respectively, in C3–4, and 9.67 MPa, 4.09 MPa, 6.75 MPa, 8.94 MPa, 6.94 MPa, and 6.13 MPa, respectively, in C6–7. While in Hirayama disease model, the maximum von Mises stress of intervertebral disc was 28.45 MPa, 10.19 MPa, 18.90 MPa, 20.70 MPa, 18.63 MPa, and 19.04 MPa, respectively, in C3–4, and 12.47 MPa, 4.28 MPa, 7.58 MPa, 9.67 MPa, 8.09 MPa, and 7.58 MPa, respectively, in C6–7 (Fig. 3).

The same rule could also be found in the C5–7 surgical segments, that was, the postoperative RoM and stress of adjacent segments under different working conditions were higher than those before operation, and the postoperative RoM and stress of patients with Hirayama disease were still higher than those of non-pathological people (Fig. 4).

### RoM and stress of adjacent segments in different PWA models

In the model with a 2-degrees decrease in PWA, the postoperative adjacent segments ROM was 17.92°, 8.49°, 19.48°, 20.65°, 17.17°and 17.15°, respectively, in C3–4, and 6.52°, 4.56°, 7.14°, 7.23°, 5.07°and 4.80°, respectively, in C6–7. While in the model with a 2-degrees increase in PWA, the postoperative adjacent segments ROM were 19.02°, 9.56°, 20.32°, 22.47°, 17.56°and 18.06°, respectively, in C3–4, and 7.02°, 4.63°, 8.32°, 8.47°, 6.56°and 6.06°, respectively, in C6–7.

The change in stress was consistent with the change in RoM, that was, either increasing or decreasing PWA, the stress increased, and in addition, the increase in stress was greater in the case of increasing PWA.

## Discussion

At present, the pathogenesis of Hirayama disease is still unclear. Many hypotheses have been put forward from the perspectives of development, genetics, immunity and so on [16–19]. Some studies have found that patients with Hirayama disease have cervical segmental instability [20], the causes of which may be due to the abnormal bony structures of the cervical spines, including smaller uncinate process and inclination angle of inferior endplate [9], and larger angle of disc-facet [8]. In this study, we modified the non-pathological human model to simulate the cervical spine structure of patients with Hirayama disease. As far as we know, this is the first time to use finite element analysis to study Hirayama disease. Our results showed that the RoM and stress of all cervical segments in patients with Hirayama disease were higher than those in non-pathological people. This indicates that the above-mentioned bony changes could cause cervical instability, thus affecting the occurrence and development of Hirayama disease.

In addition, there are many scholars who believe that the primary cause of Hirayama disease is ischaemia of the anterior horn of the spinal cord caused by sustained or repetitive flexion of the neck [21, 22]. Therefore, some doctors began to try surgical methods to treat Hirayama disease. In 2001, Imamura et al. [23] first used ACDF to treat patients with Hirayama disease (C4–C6 segments), and the follow-up 6 months after operation showed that the muscle strength of the patients recovered. This operation can achieve the purpose of treatment by fixing 2–3 segments of cervical spines so that the corresponding segments of spinal cord are no longer compressed after neck flexion. For spinal surgeons, ACDF is relatively conventional and easy to operate. For patients, ACDF has short recovery period and less trauma, so it is preferred by more surgeons. Many studies have confirmed the effectiveness of the operation. JOA score, muscle strength and electromyogram of hand internal muscle have improved significantly after ACDF [24–26].

However, the complications of ACDF should not be ignored. Although the surgical site can obtain immediate stability, the stress and RoM of adjacent segments will be increased compensatorily [27]. According to the results of our study, the RoM and stress of adjacent segments increased after ACDF in both the non-pathological and Hirayama patients. At the same time, regardless of whether the surgical segments were C4–6 or C5–7, this trend was inevitable. Notably, there are cervical hypermobility and overload in patients with Hirayama disease, so the postoperative RoM and stress of the adjacent segments are also larger; which means that although surgery can relieve the nerve compression at the lesion site, it also accelerates the degeneration of adjacent segments. Besides, we also found that, compared with the one-segment surgery [28], the adjacent segments of the two-segments surgery in this study had greater RoM and stress. It suggests that the stronger the internal fixation, the greater the possibility of adjacent segments degeneration. Our results are consistent with previous studies, ACDF may increase the risk of adjacent segment degeneration [29, 30].

Notwithstanding the degeneration of adjacent segments is the result of many factors, the increase in RoM and intervertebral disc stress after ACDF accelerates the process to some extent, which has been supported by biomechanical and imaging evidence [31–34]. In addition, some studies also show that mechanical loading has a great effect on the inner environment of the disc [35]. The increase in intervertebral disc stress will affect the intervertebral disc matrix structure, break the balance of intervertebral disc synthesis and catabolism, and then lead to disc degeneration [36]. In view of this, it is necessary for patients with Hirayama disease to exercise neck muscles and reduce head bowing after operation.

Furthermore, ACDF may change the physiological curvature of cervical spine. In this study, we increased and decreased the C4–6 PWA by 2° in our non-pathological model, with the aim of investigating the effect of different postoperative local lordosis angles on adjacent segments. From the results, only at physiological curvature was there minimum RoM and stress on adjacent segments, and in other cases, there were varying degrees of increase. This is because the change of local lordosis aggravates the hypermobility and overload of cervical spine in Hirayama disease patients. Our previous studies [37] have found that this change can also affect the postoperative outcomes of Hirayama disease patients. Hence, in order to slow down the degeneration of the adjacent segments in patients with Hirayama disease and obtain better postoperative results, the local lordosis of the cervical spine should be kept in physiological curvature as much as possible during surgery. The results of a recent study from our team on the medium—to short-term follow-up after ACDF for patients with Hirayama disease showed no statistically significant difference in the upper adjacent segment lordosis, upper adjacent RoM, and lower adjacent RoM after ACDF (*P* > 0.05) [38]. This shows that during the operation, our team reconstructed the segmental lordosis to the physiological curvature as much as possible. As for the adjacent segment degeneration of patients with Hirayama disease after ACDF, long-term follow-up is needed in the future.

There are also some limitations in this study. First of all, we recruited only one volunteer without considering the differences between healthy individuals. Secondly, there were few literature works about the changes of bony structure of Hirayama disease, and some variations may be ignored by us. Finally, we only studied some biomechanical changes after operation, and more changes need to be further studied in the future.

## Conclusion

This is the first time to use 3D finite element analysis method to investigate Hirayama disease. Our results validated that alterations in bony structures do create cervical hypermobility and overload in patients with Hirayama disease. After ACDF, patients with Hirayama disease have greater RoM and stress in adjacent segments than the non-pathological, which will accelerate the degeneration process, while maintaining the physiological curvature of the surgical segments will slow down the process.

## Data Availability

The datasets used and analysed during the current study are available from the corresponding author on reasonable request.

## Abbreviations

ACDF: Anterior cervical discectomy and fusion
RoM: Range of motion
3D: Three-dimensional
NLCS: Non-pathological lower cervical spine
LCSHD: Lower cervical spine of Hirayama disease
PWA: Posterior wall angle
